# Acute pancreatitis associated with hemorrhagic fever with renal syndrome: a cohort study of 346 patients

**DOI:** 10.1186/s12879-021-05964-5

**Published:** 2021-03-17

**Authors:** Qinyue Guo, Jing Xu, Qindong Shi, Bin Du

**Affiliations:** 1 Department of Critical Care Medicine, The First Affiliated Hospital of Xi’an Jiaotong University, 277 Yanta West Street, Xi’an, 710061 Shaanxi China; 2 Department of Emergency Medicine, The First Affiliated Hospital of Xi’an Jiaotong University, 277 Yanta West Street, Xi’an, 710061 Shaanxi China; 3 State Key Laboratory of Complex, Severe and Rare Diseases, Medical Intensive Care Unit, Peking Union Medical College Hospital, Peking Union Medical College and Chinese Academy of Medical Sciences, 1 Shuai Fu Yuan, Beijing, 100730 China

**Keywords:** Hemorrhagic fever with renal syndrome, Acute pancreatitis, Hantavirus, Propensity score match

## Abstract

**Background:**

To assess the prevalence, risk factors, clinical characteristics, and outcomes of acute pancreatitis (AP) in patients with hemorrhagic fever with renal syndrome (HFRS).

**Methods:**

All patients diagnosed with HFRS admitted to the First Affiliated Hospital of Xi’an Jiaotong University from January 2013 to July 2020 were enrolled. Patients with and without AP were compared by two risk stratification models: (1) a multivariate regression analysis using propensity score to adjust for confounding and (2) a propensity-matched nested case-control study.

**Results:**

A total of 346 patients were enrolled in the cohort study, 29 of whom (8.4%) were diagnosed as AP. There was no significant difference between patients with and without AP with regards to common risk factors and presenting signs/symptoms other than gastrointestinal symptoms (*p* < 0.01). The patients with AP had a significantly higher 90-day mortality rate (24.1% vs. 3.5%, OR 8.9, 95% CI 1.3 to 103.4, *p* = 0.045), and significantly shorter duration of therapy free-days to 28 day such as RRT and mechanical ventilation free days (*p* < 0.05, respectively).

**Conclusions:**

Our study indicated that AP was independently associated with higher mortality in HFRS patients. While considering the difficulty of early recognition of AP among HFRS patients with similar signs and/or symptoms, further laboratory and imaging studies might help identify these patients at risk of poor clinical prognosis.

**Supplementary Information:**

The online version contains supplementary material available at 10.1186/s12879-021-05964-5.

## Background

Hemorrhagic fever with renal syndrome (HFRS) is an acute infectious disease caused by Hantavirus mainly through inhalation of aerosols or dust particles contaminated by virus-containing rodent excreta [1]. Despite the widespread administration of vaccine, HFRS remains endemic in Asia and Europe, while increased incidence have been reported in China [2, 3], and selected areas of Europe, such as Germany [4].

Clinical characteristics of HFRS include hemorrhagic manifestations, renal failure, or even multiple organ dysfunction [5] Moreover, due to the lack of definitive therapy, treatment of HFRS remains supportive [6], with a mortality rate about 12% [7].

Acute pancreatitis (AP) is a rare but life-threatening complication of Hantavirus infection, with significant morbidity and mortality [8]. However, early detection of AP remains challenging, due to the high prevalence of abdominal pain (up to 64.6%) in HFRS patients even without AP [9].

Unfortunately, there have been very few reports of AP in patients with HFRS. We performed this retrospective cohort study to investigate the risk factors, clinical manifestations, laboratory abnormalities, and clinical outcomes of AP among patients with HFRS in an endemic area in China [10–12].

## Methods

### Study population

We retrospectively reviewed the medical records of all adult patients with confirmed HFRS who were admitted to the First Affiliated Hospital of Xi’an Jiaotong University from January 2013 to July 2020. The diagnosis was based on compatible clinical manifestation and laboratory detection of serum IgG antibodies using indirect immunofluorescent antibody test or serum IgM antibodies by enzyme-linked immunosorbent assay (ELISA) method. This study was approved by the institutional review board. Informed consent was waived due to the retrospective nature of the study.

### Study design

**Fig. 1:**
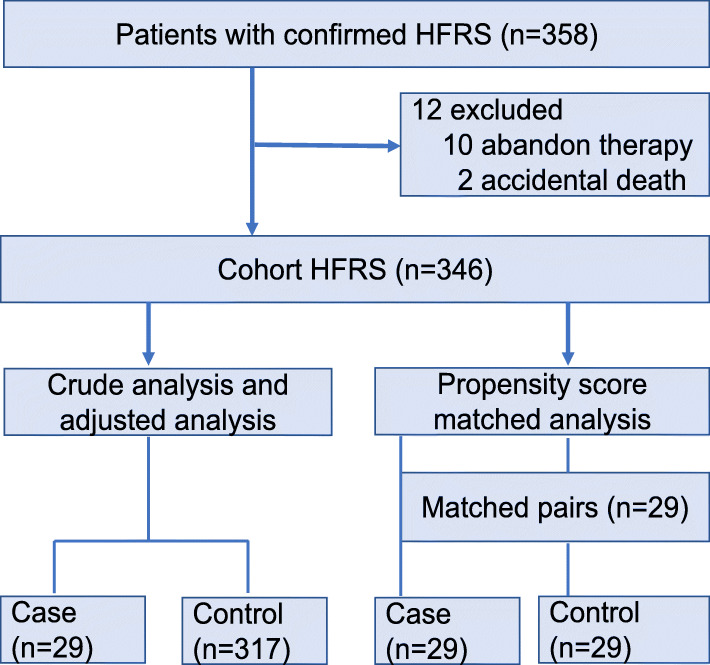
Schematic flow chart for a cohort study and matched case-control study

The first part of this study was a retrospective cohort study that included all patients with confirmed HFRS. The outcome of patients with AP was compared with that of patients without AP. The second part of the study was a matched (1:1) case-control study. For the purpose of this study, the patients with AP are designated as case subjects, and those without AP as control subjects. A schematic flow chart is shown in Fig. 1.

### Definitions

AP was diagnosed and graded according to the Atlanta criteria [13]. The detail was that the presence of two of the following three criteria: acute onset of persistent, severe, epigastric pain often radiating to the back, elevation in serum lipase or amylase to three times or greater than the upper limit of normal, and characteristic findings of acute pancreatitis on imaging (contrast-enhanced computed tomography [CT], magnetic resonance imaging [MRI], or transabdominal ultrasonography). Shock was defined as hypotension requiring administration of vasopressors to maintain mean arterial pressure (MAP) > 65 mmHg [14]. Multidrug resistant organisms (MDROs) were microorganisms that were resistant to one or more therapeutic classes of antimicrobial agents [15]. Cardiovascular diseases included coronary heart disease, hypertension, and valvular heart disease, chronic liver diseases included chronic hepatitis and cirrhosis, whereas chronic respiratory disease referred to chronic obstructive pulmonary disease, bronchitis, and interstitial lung disease.

### Data collection

Data collected included: (1) demographics (sex, age, and living habits); (2) comorbidities, such as biliary tract disease (cholecystitis, cholangitis, gallstones), diabetes, respiratory disease, tumor; (3) the course of HFRS: date of onset, symptoms at presentation, date of hospital admission, and complications (such as shock and MDROs infection); (4) laboratory findings on hospital admission: complete blood count, blood chemistry, and inflammatory biomarkers (C-reactive protein [CRP], procalcitonin [PCT]), (5) treatment: intensive care unit (ICU) admission, mechanical ventilation, renal replacement therapy (RRT); and (6) outcome measures: 90-day all-cause mortality rate, and hospital-free days at day 28.

### Statistical analysis

Categorical variables were examined by Fisher exact test or chi-square test, as appropriate, while continuous variables were compared by student t test or Mann-Whitney U test. All tests of significance were two-tailed and a *P* value of 0.05 was considered significant.

In order to account for potential confounding factors in this observational study, we developed a propensity score, using multivariate logistic regression analysis without regards to outcomes [16, 17], to adjust for the differences in baseline characteristics between HFRS patients with and without AP. All prespecified covariates (age, sex, smoker, alcoholics, whether there are comorbidities, days from symptom onset to hospitalization, whether there are gastrointestinal symptoms), as outlined above, were included in the final prediction model for AP among HFRS patients, by means of stepwise backward elimination with *p* value < 0.1. Model discrimination was assessed by area under the receiver operating characteristics curve (AUROC).

The effect of AP on 90-day mortality, as well as other clinical outcomes (i.e. ICU admission, RRT, mechanical ventilation, MDRO infection), was analyzed by stepwise backward logistic regression model by including any covariate with *p* value < 0.10 in univariate analysis. Moreover, individual propensity score was also included in the model as a covariable to calculate the adjusted odds ratios (aOR) and 95% confidence intervals (CIs). In addition, we performed a nested case-control study (1:1 match) by matching case and control subjects using calipers of width equal to 0.2 of the standard deviation of the logit of the propensity score [18, 19]. A nearest-neighbor matching algorithm was employed to form pairs of case and control subjects, once a match was made, previous matches were not reconsidered before making the next match. Survival curves for case and control subjects were analyzed by the Kaplan-Meier method and compared by log-rank test.

Results were analyzed with SPSS version 22.0 K for Windows (SPSS Inc., Chicago, IL, USA) and STATA SE 14 (StataCorp LP, College Station, TX, USA).

## Results

### Patient enrolment and clinical characteristics

During the study period, 358 patients with the diagnosis of HFRS were admitted to the First Affiliated Hospital of Xi’an Jiaotong University. After exclusion of 12 patients due to withholding or withdrawal of life-sustaining therapies or accidental death, 346 patients (mean age 46.1, 255 men [73.7%]) were included in the final analysis (Fig. 1).

One hundred and forty-seven patients had comorbidities, such as cardiovascular disease (*n* = 45, 13.0%), diabetes (*n* = 25, 7.2%), chronic liver disease (*n* = 24, 6.9%), biliary tract disease (*n* = 11, 3.2%), and others. The most common presenting signs and symptoms included fever (*n* = 302, 87.3%), back/flank pain (*n* = 213, 61.6%), gastrointestinal symptoms (*n* = 118, 34.1%), and shock (*n* = 33, 9.5%). Of 118 patients with presenting gastrointestinal symptoms, 45 had abdominal pain, 13 had nausea/vomiting, 48 had diarrhea, and 12 had abdominal distention. RRT and mechanical ventilation were used in 86 (24.9%) and 17 (4.9%) patients, respectively. A total of 25 patients (7.2%) were admitted to ICU, and 14 patients (4.0%) died at 90 days.

### Patients with acute pancreatitis and development of propensity score

A total of 29 patients (8.4%) were diagnosed as AP, including 19 patients having AP on hospital admission, and 10 patients developing AP during hospitalization. Compared with HFRS patients without AP, patients with AP were more likely to be smokers and alcoholics (Table 1). There was no significant difference between patients with and without AP with regards to common risk factors (biliary tract disease, diabetes and other underlying diseases) and presenting signs/symptoms (fever, back/flank pain, and shock) other than gastrointestinal symptoms (*p* < 0.01) (Table 1).

**Table 1:** Baseline characteristics and clinical outcome of hemorrhagic fever renal syndrome patients with and without acute pancreatitis

	Case (*n* = 29)	Control (*n* = 317)	*P* value
Age, year (mean ± SD)	44.4 ± 16.4	46.2 ± 17.3	0.63
Male sex	19 (65.5%)	236 (74.4%)	0.32
Smoker	11 (37.9%)	132 (41.6%)	**0.02**
Alcoholics	9 (31.0%)	40 (12.6%)	**0.03**
Comorbidities			
No comorbidities	14 (48.3%)	185 (58.4%)	0.33
Cardiovascular disease	6 (20.7%)	39 (12.3%)	0.24
Diabetes	2 (6.9%)	23 (7.3%)	> 0.99
Chronic liver disease	2 (6.9%)	22 (6.9%)	> 0.99
Biliary tract disease	3 (10.3%)	8 (2.5%)	0.06
Chronic respiratory disease	2 (6.9%)	9 (2.8%)	0.23
Pregnancy	1 (3.5%)	4 (1.3%)	0.36
Chronic renal disease	0 (0)	4 (1.3%)	> 0.99
Solid tumor	0 (0)	2 (0.6%)	> 0.99
Others	2 (6.9%)	37 (11.7%)	0.76
Days from symptom onset to hospitalization, d (mean ± SD)	4.6 ± 2.6	6.4 ± 5.4	**0.02**
Presenting symptoms			
Fever	24 (82.8%)	278 (87.7%)	0.39
Back/Flank pain	14 (48.3%)	199 (62.8%)	0.16
Gastrointestinal symptoms	20 (69.0%)	98 (30.9%)	**< 0.01**
Nausea/vomiting	2 (6.9%)	11 (3.5%)	0.30
Abdominal pain	8 (27.6%)	37 (11.7%)	**0.04**
Diarrhea	7 (24.1%)	41 (12.9%)	0.09
Abdominal distention	3 (10.3%)	9 (2.8%)	0.06
Shock	5 (17.2%)	28 (8.8%)	0.18
Treatment			
Antibiotic therapy (mean ± SD)	1.7 ± 1.2	0.7 ± 0.8	**0.01**
ICU admission	13 (44.8%)	12 (3.8%)	**< 0.01**
ICU-free days at day 28, d (mean ± SD)	19.1 ± 10.2	27.1 ± 4.8	**< 0.01**
RRT	17 (58.6%)	69 (21.8%)	**< 0.01**
RRT-free days at day 28, d (mean ± SD)	18.6 ± 11.4	25.2 ± 6.5	**< 0.01**
Mechanical ventilation	9 (31.0%)	8 (2.5%)	**< 0.01**
Ventilator-free days at day 28, d (mean ± SD)	21.3 ± 11.5	27.4 ± 4.1	**< 0.01**
Clinical outcome			
90-day mortality	7 (24.1%)	7 (2.2%)	**< 0.01**
Hospital-free days at day 28, d (mean ± SD)	8.6 ± 8.4	16.4 ± 6.6	**0.005**

*ICU* Intensive care unit, *RRT* Renal replacement therapy

Data are presented as the number (percentage) of patients unless indicated otherwise

On hospital admission, compared with patients without AP, patients with AP were characterized by more pronounced inflammation (as suggested by higher white cell count, higher CRP), lower platelet count, hypocalcemia, and abnormal liver functions (as suggested by higher liver enzymes, higher bilirubin, and lower albumin) (Table 2). In addition, patients in both groups suffered from mild to moderate renal dysfunction, as indicated by elevated serum creatinine and BUN levels. Interestingly, there was no significant difference between the two groups with regards to serum lipase and serum/urine amylase levels on hospital admission (Table 2).

**Table 2:** Laboratory results on hospital admission in hemorrhagic fever renal syndrome patients with and without acute pancreatitis

	Case (n = 29)	Control (n = 317)	*P* value
WCC (× 10^9^/L)	20.0 ± 15.2	11.9 ± 9.1	**0.01**
Platelet (× 10^9^/L)	55.5 ± 84.0	110.3 ± 98.1	**0.01**
Neutrophil (%)	76.9 ± 27.2	63.9 ± 19.2	**0.01**
C-reactive protein (mg/L)	45.8 ± 31.4	30.9 ± 19.5	**0.02**
Procalcitonin (ng/ml)	12.9 ± 37.0	4.8 ± 11.8	0.20
Albumin (g/L)	29.5 ± 5.6	34.8 ± 7.0	**0.01**
Total bilirubin (μmol/L)	15.6 ± 6.9	11.2 ± 5.3	**0.01**
AST (U/L)	194.8 ± 261.6	67.2 ± 100.8	**0.01**
ALT (U/L)	83.0 ± 84.6	42.9 ± 24.5	**0.02**
ALP (U/L)	78.67 ± 53.9	99.7 ± 32.8	0.75
LDH (U/L)	1056 ± 1150	547.9 ± 280.8	**0.04**
Triglyceride (mmol/L)	3.21 ± 1.91	9.89 ± 75.2	0.93
Low density lipoprotein (mmol/L)	1.44 ± 1.05	1.8 ± 0.6	0.29
High-density lipoprotein (mmol/L)	0.6 ± 0.2	0.9 ± 0.8	0.14
Serum creatinine (μmol/L)	292.2 ± 260.6	217.5 ± 205.8	0.14
BUN (mmol/L)	15.1 ± 10.7	11.7 ± 9.0	0.12
Serum calcium (mmol/L)	1.8 ± 0.2	2.0 ± 0.2	**0.01**
Serum amylase (U/L)	257.9 ± 232.4	170.7 ± 97.8	0.39
Urine amylase (U/L)	200.2 ± 194.1	133 ± 26.1	0.65
Serum lipase (U/L)	1261.0 ± 1068.2	988.2 ± 899.1	0.39

*ALT* Alanine aminotransferase, *ALP* Alkaline phosphatase, *AST* Aspartate aminotransferase, *BUN* Blood urea nitrogen, *LDH* Lactate dehydrogenase, *WCC* White blood cell count

Data are presented as the mean ± SD

Based on above data, we developed a propensity score using a multivariate logistic regression model with an area under the receiver operating characteristics curve of 0.71 (95%CI 0.62 to 0.81) (Supplementary material Figure), indicating good discrimination of HFRS patients developing AP.

### Acute pancreatitis as risk factor for clinical outcome

Compared with patients without AP, patients with AP were more severe, as indicated by more antibiotic combination therapy, more life-sustaining therapies (i.e. mechanical ventilation, RRT), more ICU admissions (44.8% vs. 3.8%, OR 20.7, 95%CI 8.4 to 53.5, *p* < 0.01), and shorter hospital-free days, as well as higher 90-day mortality rate (24.1% vs. 2.2%, OR 14.1, 95%CI 4.8 to 40.4, *p* < 0.01) (Tables 1 and 3).

**Table 3:** Analysis of acute pancreatitis associated with 90-day mortality rate and supportive treatment in 346 patients with hemorrhagic fever renal syndrome

	Crude		Adjusted		Propensity-matched^d^	
	OR (95% CI)	*P* Value	OR (95% CI)	*P* Value	OR (95% CI)	*P* Value
90-day mortality	14.1 (4.8–40.4)	**< 0.01**	17.6^a^ (4.3–73.3)	**< 0.01**	8.9 (1.3–103.4)	**0.045**
ICU admissions	20.7 (8.4–53.5)	**< 0.01**	22.8^a^ (7.8–66.4)	**< 0.01**	22.8 (3.3–249.6)	**< 0.01**
RRT	5.1 (2.3–11.3)	**< 0.01**	4.3^b^ (1.9–9.6)	**< 0.01**	3.2 (1.1–8.7)	0.06
Mechanical ventilation	17.4 (6.1–52.4)	**< 0.01**	32.5^a^ (7.9–134.4)	**< 0.01**	12.6 (1.64–142.4)	**0.01**
MDRO infection	7.8 (1.3–38.8)	0.06	5.02^c^ (0.5–46.9)	0.16	2.07 (0.2–31.0)	> 0.99

*CI* Confidence interval, *OR* Odds ratio, *ICU* Intensive care unit, *RRT* Renal replacement therapy, *MDRO* Multiple drug resistant organism

Adjusted for variables (^a^alcoholics; ^b^ alcoholics, days from symptom onset to hospitalization; ^c^alcoholics, days from symptom onset to hospitalization, gastrointestinal symptoms) associated with 90-day mortality, treated in ICU, treated with CRRT, treated with mechanical ventilation, infected with MDRO and the propensity score of each patient’s likelihood of being diagnosed with acute pancreatitis

^d^Of 346 patients, 29 pairs were matched

In multivariate regression analysis adjusted for potential confounders (smoker, alcoholics, whether there are comorbidities, days from symptom onset to hospitalization, whether there are gastrointestinal symptoms) with propensity score, AP was associated with increased 90-day mortality rate (adjusted odds ratio [aOR], 17.6, 95% CI, 4.3 to 73.3, *p* < 0.01). In addition, AP was an independent risk factor of ICU admission (aOR, 22.8; 95% CI, 7.8 to 66.4, *p* < 0.01), mechanical ventilation (aOR, 32.5; 95% CI, 7.9 to 134.4, *p* < 0.01), and RRT (aOR, 4.3; 95% CI, 1.9 to 9.6, *p* < 0.01) (Table 3).

### Matched case–control study

**Fig. 2:**
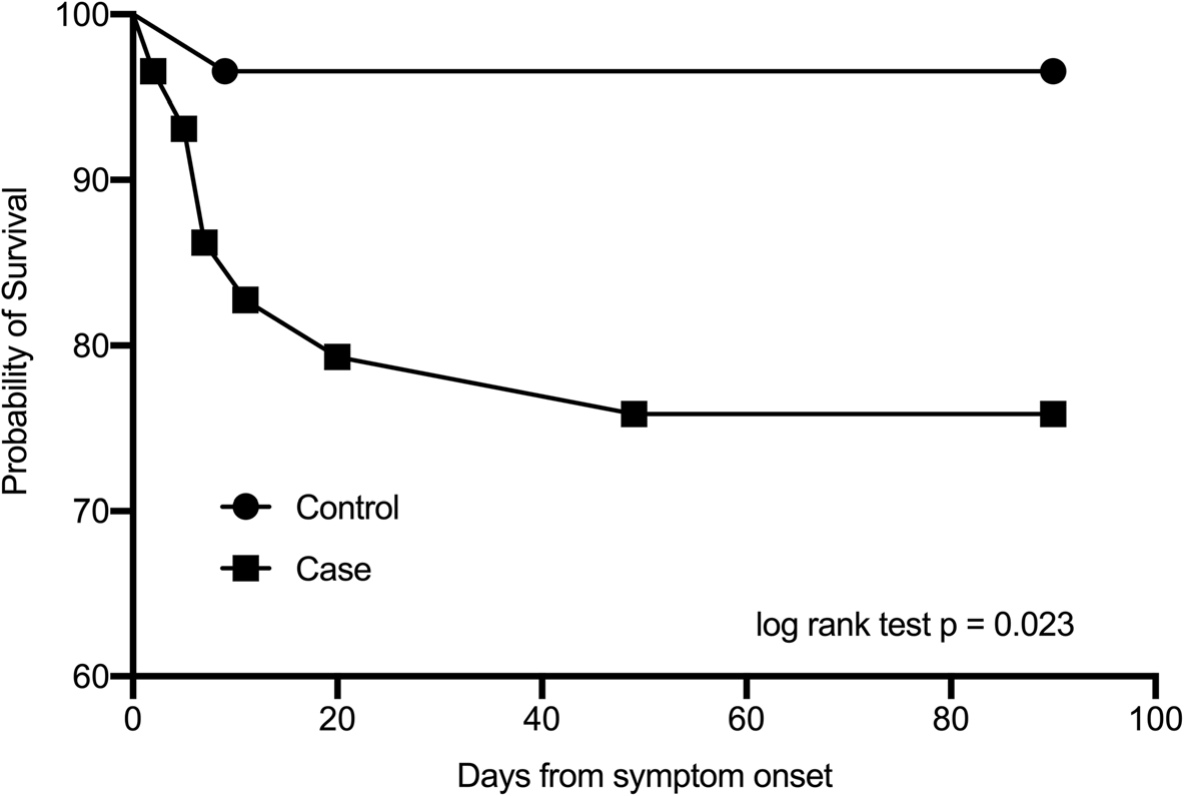
Kaplan-Meier survival curve of 29 pairs of HFRS patients stratified by the presence of acute pancreatitis. HFRS, hemorrhagic fever with renal syndrome

In propensity score-matched case-control study, 29 HFRS patients with AP (case subjects) were successfully matched with 29 HFRS patients without AP (control subjects) (Supplementary material Table 1). Case subjects were more likely to receive mechanical ventilation (OR, 12.6; 95% CI, 1.64 to 142.4, *p* = 0.01), ICU admission (OR, 22.8; 95% CI, 3.3 to 249.6, *p* < 0.01), but not RRT (OR, 3.2; 95% CI, 1.1 to 8.7, *p* = 0.06) (Supplementary material Table 2). Compared with control subjects, case subjects had a significantly higher 90-day mortality rate (24.1% vs. 3.5%, OR 8.9, 95% CI 1.3 to 103.4, *p* = 0.045) (Table 3, and Fig. 2), and shorter RRT-free days and ventilator-free days (both *p* < 0.05) (Supplementary material Table 2).

## Discussion

Diverse clinical manifestations had been reported in HFRS patients, ranging from mild and acute influenza-like illness to more severe shock syndrome. Apart from acute renal insufficiency, up to one-third of HFRS patients exhibited extrarenal organ involvement, with pancreatobiliary diseases as the most common manifestation, including acalculous cholecystitis, pancreatitis, and cholangitis [9]. Hullinghorst and Steer reported pathological evidence of pancreatitis in one-third of HFRS autopsies during the Korean conflict [20]. The reported prevalence of AP among HFRS patients was highly variable in observational studies, ranging from 2.8 to 78%, with a pooled prevalence of 6.8% (36/529) [9, 21–25]. We found that 8.4% of HFRS patients in our cohort developed AP. The observed difference in prevalence of AP among HFRS patients might be related, at least in part, to the causative viruses [9], geographic region [24], male-to-female ratio [8], prevalence of risk factors (such as alcohol misuse and history of gallstones), and time course during disease progression. For example, the highest prevalence of AP was reported by Bui-Mansfield and colleagues in a group of 13 male patients with HFRS [22], whereas the lowest prevalence was reported by Zhu and coworkers in 218 HFRS patients (150 males and 68 females) [23].

Another major finding of our study was that AP was an independent risk factor for 90-day mortality in HFRS patients, which had never been studied in the above-mentioned observational studies, possibly due to the limited number of AP cases (3 to 12) in the individual study [8, 9, 21–25]. However, pooled results from these studies suggested similar mortality rates [8.3% (3/36) in HFRS patients with AP vs 4.9% (24/493) in HFRS patients without AP, *p* = 0.3618]. In comparison, the 29 cases in our cohort represented the largest number of AP cases among HFRS patients ever reported, which allowed us to investigate the impact of AP on mortality in univariate and multivariate analyses. The significantly higher mortality rate in HFRS patients with AP (24.1% vs. 2.2%) could be explained by the severity of acute illness, as demonstrated by more pronounced inflammation (higher white cell count and CRP levels), liver dysfunction, more life-sustaining therapies (including mechanical ventilation and RRT), and more ICU admissions (44.8% vs. 3.8%).

It is a common belief that early recognition of patients with AP might be very important to improve clinical outcome of this potentially life-threatening condition. However, early diagnosis of AP among HFRS patients might be difficult, as both diseases shared some common clinical signs/symptoms, such as nausea/vomiting and abdominal pain. For example, abdominal pain was a presenting symptom in 30 to 90% of HFRS patients, which might explain the observed high misdiagnosis rate (up to 90%) [8]. As a result, HFRS patients with abdominal pain should be subject to follow-up laboratory (i.e. pancreatic and/or liver enzymes) and imaging (i.e. abdominal CT scan or ultrasonography) investigations [9, 24], in order to determine the presence and severity of pancreatobiliary complications.

The major strength of our study was the robustness of the study result (i.e. AP as an independent risk factor for mortality), which was supported by univariate analysis, multivariate regression analysis adjusted for propensity score, and propensity score-matched case-control analysis. Our study was also subject to limitations. First, this was a retrospective single-center study, the result of which might not be generalized to other settings and required further validation by prospective multicenter studies. Nonetheless, the number of AP cases as well as HFRS patients in our cohort was significantly higher than that in previous studies. Second, the prevalence of AP might be underestimated, because laboratory (serum amylase or lipase) and abdominal imaging investigations were only performed in selected but not all HFRS patients. Last, we only compared laboratory tests on hospital admission, when only 19 out of 29 patients with AP were diagnosed as such, in whom the baseline laboratory tests at AP onset were not available.

That may explain why there was no difference in serum lipase and serum/urinary amylase of two groups.

## Conclusions

In conclusion, our study indicated that AP was independently associated with higher mortality in HFRS patients. While considering the difficulty of early recognition of AP among HFRS patients with similar signs and/or symptoms, further laboratory and imaging studies might help identify these patients at risk of poor clinical prognosis.

## Supplementary Information

**Additional file 1.**

## Data Availability

The datasets analyzed during the current study are available from the corresponding author upon reasonable request.

## Abbreviations

AP: Acute pancreatitis
HFRS: Hemorrhagic fever with renal syndrome
ELISA: Enzyme-linked immunosorbent assay
CT: Computed tomography
MRI: Magnetic resonance imaging
MDROs: Multidrug resistant organisms
CRP: C-reactive protein
PCT: Procalcitonin
ICU: Intensive care unit
RRT: Renal replacement therapy
AUROC: Area under the receiver operating characteristics curve
CI: Confidence interval
OR: Odds ratio
ALT: Alanine aminotransferase
ALP: Alkaline phosphatase
AST: Aspartate aminotransferase
BUN: Blood urea nitrogen
LDH: Lactate dehydrogenase
WCC: White blood cell count
