# Supplementary material for: Acute pancreatitis associated with hemorrhagic fever with renal syndrome: a cohort study of 346 patients
Source: BMC Infect Dis. 2021 Mar 17;21:267. doi: 10.1186/s12879-021-05964-5 (PMC7967104; doi:10.1186/s12879-021-05964-5)
Supplement: Supplementary file 1 — Additional file 1. [file 12879_2021_5964_MOESM1_ESM.docx]

Acute Pancreatitis Associated with Hemorrhagic Fever with Renal Syndrome:

A Cohort Study of 346 Patients

Supplementary Material

Figure. Receiver operating characteristics (ROC) curve for the propensity score for a HFRS patient’s likelihood of diagnosed with acute pancreatitis. AUC, area under ROC curve.

Table 1. Baseline characteristics of HFRS patients with and without acute pancreatitis in propensity score-matched case-control study

|  | Case  (n = 29) | Control  (n = 29) | Standardized difference |
| --- | --- | --- | --- |
| Propensity score (mean±SD) | 0.1±0.1 | 0.1±0.08 | 0.01 |
| Age, yr (mean±SD) | 44.4±16.4 | 45.3±18.1 | 0.047 |
| Male sex | 19(65.5%) | 18 (62.1%) | 0.07 |
| Smoker | 11(37.9%) | 8 (27.6%) | 0.22 |
| Alcoholics | 9 (31.0%) | 7 (24.1%) | 0.15 |
| Comorbidities |  |  |  |
| No comorbidities | 14 (48.3%) | 15 (51.7%) | 0.07 |
| Cardiovascular disease | 6 (20.7%) | 5 (17.2%) | 0.09 |
| Diabetes | 2 (6.9%) | 1 (3.4%) | 0.16 |
| Chronic liver disease | 2 (6.9%) | 5 (17.2%) | 0.32 |
| Biliary tract disease | 3 (10.3%) | 3 (10.3%) | 0.01 |
| Chronic respiratory disease | 2 (6.9%) | 1 (3.4%)) | 0.16 |
| Pregnancy | 1 (3.4%) | 0 (0%) | 0.27 |
| Chronic renal disease | 0 (0%) | 1 (3.4%) | 0.27 |
| Solid tumor | 0 (0%) | 0 (0%) | 0.00 |
| Others | 2 (6.9%) | 2 (6.9%) | 0.00 |
| Days from symptom onset to hospitalization, d (mean±SD) | 4.6±2.6 | 4.5±2.1 | 0.06 |

HFRS, hemorrhagic fever with renal syndrome

Data are presented as the number (percentage) of patients unless indicated otherwise.

Table 2. Clinical outcomes of HFRS patients with and without acute pancreatitis in propensity score-matched case-control study

|  | Case  (n = 29) | Control  (n = 29) | P value |
| --- | --- | --- | --- |
| Treatment |  |  |  |
| Duration of antibiotic therapy (mean±SD) | 1.7±1.2 | 0.9±0.8 | **<0.01** |
| ICU admission | 13 (44.8%) | 1 (3.5%) | **<0.01** |
| ICU-free days at day 28, d (mean±SD) | 19.1±10.2 | 27.0±5.2 | **<0.01** |
| RRT | 17 (58.6%) | 9 (31.0%) | 0.06 |
| RRT-free days at day 28, d (mean±SD) | 18.6±11.4 | 23.8±8.4 | **0.04** |
| Mechanical ventilation | 9 (31.0%) | 1 (3.5%) | **0.01** |
| VFDs at day 28, d (mean±SD) | 21.3±11.5 | 27.0±5.2 | **<0.01** |
| Clinical outcome |  |  |  |
| 90-day mortality | 7 (24.1%) | 1 (3.5%) | **0.045** |
| Hospital-free days at day 28, d (mean±SD) | 8.6±8.4 | 15.3±7.2 | **<0.01** |

HFRS, hemorrhagic fever with renal syndrome; ICU, intensive care unit; RRT, renal replacement therapy; VFD, ventilator-free day.

Data are presented as the number (percentage) of patients unless indicated otherwise.
